# Increased circulating level of interleukin-6 and CD8^+^ T cell exhaustion are associated with progression of COVID-19

**DOI:** 10.1186/s40249-020-00780-6

**Published:** 2020-11-25

**Authors:** Peng-Hui Yang, Yi-Bo Ding, Zhe Xu, Rui Pu, Ping Li, Jin Yan, Ji-Luo Liu, Fan-Ping Meng, Lei Huang, Lei Shi, Tian-Jun Jiang, En-Qiang Qin, Min Zhao, Da-Wei Zhang, Peng Zhao, Ling-Xiang Yu, Zhao-Hai Wang, Zhi-Xian Hong, Zhao-Hui Xiao, Qing Xi, De-Xi Zhao, Peng Yu, Cai-Zhong Zhu, Zhu Chen, Shao-Geng Zhang, Jun-Sheng Ji, Fu-Sheng Wang, Guang-Wen Cao

**Affiliations:** 1 Department of Hepatobiliary, The Fifth Medical Center of PLA General Hospital, National Clinical Research Center for Infectious Diseases, Beijing, 100039 China; 2 Treatment and Research Center for Infectious Diseases, The Fifth Medical Center of PLA General Hospital, National Clinical Research Center for Infectious Diseases, Beijing, 100039 China; 3 Department of Epidemiology, Second Military Medical University, Shanghai, 200433 China

**Keywords:** COVID-19, Progression, Interleukin-6, CD8^+^ T cell exhaustion, Prospective case series

## Abstract

**Background:**

Coronavirus disease 2019 (COVID-19) is pandemic. It is critical to identify COVID-19 patients who are most likely to develop a severe disease. This study was designed to determine the clinical and epidemiological features of COVID-19 patients associated with the development of pneumonia and factors associated with disease progression.

**Methods:**

Seventy consecutive patients with etiologically confirmed COVID-19 admitted to PLA General Hospital in Beijing, China from December 27, 2019 to March 12, 2020 were enrolled in this study and followed-up to March 16, 2020. Differences in clinical and laboratory findings between COVID-19 patients with pneumonia and those without were determined by the *χ*^2^ test or the Fisher exact test (categorical variables) and independent group *t* test or Mann–Whitney U test (continuous variables). The Cox proportional hazard model and Generalized Estimating Equations were applied to evaluate factors that predicted the progression of COVID-19.

**Results:**

The mean incubation was 8.67 (95% confidence interval, 6.78–10.56) days. Mean duration from the first test severe acute respiratory syndrome coronavirus 2-positive to conversion was 11.38 (9.86–12.90) days. Compared to pneumonia-free patients, pneumonia patients were 16.5 years older and had higher frequencies of having hypertension, fever, and cough and higher circulating levels of neutrophil proportion, interleukin-6, low count (< 190/µl) of CD8^+^ T cells, and neutrophil/lymphocyte ratio. Thirteen patients deteriorated during hospitalization. Cox regression analysis indicated that older age and higher serum levels of interleukin-6, C-reactive protein, procalcitonin, and lactate at admission significantly predicted the progression of COVID-19. During hospitalization, circulating counts of T lymphocytes, CD4^+^ T cells, and CD8^+^ T cells were lower, whereas neutrophil proportion, neutrophil/lymphocyte ratio, and the circulating levels of interleukin-6, C-reactive protein, and procalcitonin were higher, in pneumonia patients than in pneumonia-free patients. CD8^+^ lymphocyte count in pneumonia patients did not recover when discharged.

**Conclusions:**

Older age and higher levels of C-reactive protein, procalcitionin, interleukin-6, and lactate might predict COVID-19 progression. T lymphocyte, especially CD8^+^ cell-mediated immunity is critical in recovery of COVID-19. This study may help in predicting disease progression and designing immunotherapy for COVID-19.

## Background

Coronavirus disease 2019 (COVID-19) caused by severe acute respiratory syndrome coronavirus 2 (SARS-CoV-2) is being pandemic in 2020 [1–4]. As of November 6, 2020, a total of 48 534 508 cases and 1 231 017 deaths were reported globally [5]. Clinical course of SARS-CoV-2 differs greatly, ranging from asymptomatic and mild symptoms including hyposmia to fatal pneumonia [4, 6]. Approximately 80% of COVID-19 cases have mild disease, 20% require hospital admission, and 5% require intensive care admission [7]. Approximately, 31.25–66.67% of early COVID-19 cases do not have radiographic pneumonia, pneumonia detected by radiographic image [8, 9]. It is critical to identify COVID-19 patients who are most likely to have poor outcomes [10]. Differences in the epidemiological and clinical features between COVID-19 patients with pneumonia and those without are initiatory to recognize patients who are more likely to develop progressive diseases. To determine the factors that predict the progression of COVID-19 in a prospective study is critical for the development of suitable prophylactic options. However, these prognostic factors remain largely unknown. Furthermore, the course of COVID-19 and the duration of viral shedding, which reflect the interaction of SARS-CoV-2 with host immunity, remain to be further clarified. Here, we report a series of cases admitted into an infectious disease hospital that is responsible for quarantine and treatment of COVID-19 assigned by the government of Beijing, China.

## Methods

### Patients enrollment

This case series was approved by the institutional ethics board of Fifth Medical Center of PLA General Hospital, with orally informed consents. All consecutive patients with COVID-19 admitted to the Fifth Medical Center of PLA General Hospital from December 27, 2019 to March 12, 2020 were enrolled. Patients with suspected COVID-19 were admitted to this center and quarantined. Respiratory samples including throat swab samples were detected for the presence of SARS-CoV-2 by quantitative real-time reverse transcription polymerase chain reaction (RT-PCR) as previously described [1, 2, 4]. Epidemiological information was obtained from person-to-person interview for exposure history in the past 2–3 weeks. Incubation duration was calculated in the cases with clear time points of exposure and illness onset. Clinical, laboratory, and radiographic characteristics as well as therapeutic activities and outcomes were collected. Pneumonia was diagnosed by chest computerized tomography scan. All patients received blood test at the 1st day when admitted into the study hospital. The test was mostly repeated every 2–4 days during hospitalization according to patient’s condition or every day if COVID-19 deteriorated. The total leukocyte and percentages of neutrophils and lymphocyte in peripheral blood were measured on an automatic hematology analyzer HN-2000 series (SYSMEX, Kobe, Japan). Serum C-reactive protein (CRP), glucose, triglyceride, and lactate were measured on an automatic biochemical analyzer AU5400 (BECKMAN COULTER, Brea, CA). Interleukin-6 (IL-6) and procalcitonin were examined using Roch cobas 8000 (Roche Disgnostics GmbH, Mannheim, GER). Erythrocyte sedimentation rate (ESR) was measured by an automatic ESR analyzer VACUETTE (Greiner Bio-One, Kremsmünster, Austria). Lymphocyte count and subsets were measured using a cytometry FACSCalibur (BD Biosciences, Becton, NJ). Parameters of respiration were measured using a blood gas analyzer OPTI CCA-TS (OPTI Medical Systems, Roswell, GA). COVID-19 was re-diagnosed according to the Protocol for the Diagnosis and Treatment of COVID-19 (Version 7th), National Health Commission of the People’s Republic of China [11]. Briefly, the diagnosis of COVID-19 was confirmed if patient’s respiratory sample was positive for SARS-CoV-2 genomic RNA. All diagnosed COVID-19 patients were classified as mild, common, severe, and extremely severe types. Mild type was defined if COVID-19 patient had mild symptoms but did not have radiographic pneumonia. Common type was identified if COVID-19 patient had fever, respiratory symptoms, and radiographic pneumonia. Severe type was diagnosed if COVID-19 patient with pneumonia met one of the following criteria: respiratory rate ≥ 30 times/min, resting oxygen saturation ≤ 93%, and arterial partial pressure of oxygen (PaO_2_)/fraction of inspired oxygen (FiO_2_) ≤ 300 mmHg. Extremely severe type met one of the following criteria: respiratory failure that needs mechanical ventilation, shock, and combined organ failure that need to be treated in intensive care unit. Patients were discharged from hospital if they met the criteria: temperature normalized for ≥ 3 days, apparent improvement in respiratory symptoms, absorption of pneumonia, and negative SARS-CoV-2 genomic RNA tested for twice at 1 day interval. The final date of follow-up was March 16, 2020.

### Factors associated with the presence of pneumonia and COVID-19 progression-predicting factors

Categorical variables were compared using the *χ*^2^ test or the Fisher exact test. Continuous variables were described using mean, median, and interquartile range (IQR) values and compared using independent group *t* test or Mann–Whitney U test. The Cox proportional hazard model was applied to calculate the hazard ratio (HR) and 95% confidence interval (*CI*). Due to the small sample size, the analyses were not adjusted for multiple comparisons, given the potential for type I error. Differences in the daily medians of laboratory parameters between patients with and without pneumonia in the 20 consecutive days were evaluated using Generalized Estimating Equations (GEE). Above statistical analyses were two-sided and performed using Statistical Package for the Social Sciences (SPSS, IBM Corp, Armonk, NY) version 21.0 software. The dynamic diagrams and the trend of each laboratory parameter fitted by local polynomial regression were generated by R software (version 3.6.2, R Foundation for Statistical Computing, Canberra, Austria). A *P* value of < 0.05 was considered significant.

## Results

### Epidemiological characteristics

Seventy consecutive patients with laboratory-confirmed COVID-19 were enrolled in this study. Of those, 33 (47.1%) had direct exposure and 24 (34.3%) had an indirect exposure to Wuhan within 14 days before disease onset. The patients included two family clustering cases (Additional file 1: Fig. 1). Confirmed cases kept increasing since January 10, reached the peak on January 22, 2020. Ten days after the Wuhan quarantine, the curve touched the bottom. Thereafter, 3 cases came from other countries (Additional file 2: Fig. 2). The mean (95% *CI*) incubation period of COVID-19 was 8.67 (6.78–10.56) days in 42 cases with clear time points of exposure and illness onset. The incubation was 8.35 (5.15–11.56) days in patients without pneumonia and 8.88 (6.37–11.39) days in patients with pneumonia.

Of the 70 patients, 62 were discharged. One co-infected with human immunodeficiency virus (HIV) and hepatitis B virus (HBV) was excluded from the analysis because of known aberrant immunity. The mean (95% *CI*) duration from the first SARS-CoV-2-positive test to RT-PCR conversion in the remaining 61 cases was 11.38 (9.86–12.90) days.

### Clinical and laboratory parameters of COVID-19 patients with pneumonia and those without when just hospitalized

The median age was 45.0 years (IQR: 34.5–61.0; range: 3–85 years), and 32 (45.7%) were women. Major symptoms at disease onset were fever (84.3%), cough (51.4%), fatigue (28.6%), expectoration (22.9%), sore throat (20.0%), and myalgia (18.6%). Of the 70 patients, 38 (54.3%) had at least one coexisting health conditions. Hypertension (24.3%) and diabetes (10.0%) were the most common ones.

The numbers of mild, common, severe, and extremely severe patients identified at the time of being admitted were 22, 32, 15, and 1, respectively. Common, severe, and extremely severe types were combined as COVID-19 patients with pneumonia. Compared to patients without pneumonia, patients with pneumonia were 16.5 years older and had higher rates of hypertension, fever, and cough and longer fever duration (Table 1). Laboratory examinations showed that pneumonia patients had higher circulating levels of neutrophils proportion, IL-6, very low count (< 190/µl) of CD8^+^ T cells, and neutrophil/lymphocyte ratio (NLR) than did pneumonia-free COVID-19 patients at the 1st day after admission to hospital (Table 2).

**Table 1:** Baseline information of COVID-19 patients enrolled in this study

	All enrolled patients (*n* = 70)	Patients without pneumonia (*n* = 22)	Patients with pneumonia (*n* = 48)	*P*
Age, median (IQR)—years	45.00 (34.50–61.00)	34.50 (21.75–44.00)	51.00 (39.00–67.00)	** < 0.001**
Gender				0.314
Male	38 (54.3)	14 (63.6)	24 (50.0)	
Female	32 (45.7)	8 (36.4)	24 (50.0)	
BMI, median (IQR)	24.63 (22.33–26.50)	25.03 (21.40–27.46)	24.44 (22.34–26.34)	0.979
Exposure				**0.002**
No explicit contact	13 (18.6)	0	13 (27.1)	
Wuhan-direct	33 (47.1)	16 (72.7)	17 (35.4)	
Wuhan-indirect	24 (34.3)	6 (27.3)	18 (37.5)	
Underlying diseases				
Hypertension	17 (24.3)	1 (4.5)	16 (33.3)	**0.009**
Diabetes	7 (10.0)	2 (9.1)	5 (10.4)	1.000
Main symptoms				
Fever	59 (84.3)	15 (68.2)	44 (91.7)	**0.029**
Cough	36 (51.4)	7 (31.8)	29 (60.4)	**0.026**
Sore throat	14 (20.0)	5 (22.7)	9 (18.8)	0.752
Sputum production	16 (22.9)	3 (13.6)	13 (27.1)	0.214
Diarrhea	5 (7.1)	0	5 (10.4)	0.173
Fatigue	20 (28.6)	4 (18.2)	16 (33.3)	0.193
Myalgia	13 (18.6)	4 (18.2)	9 (18.8)	1.000
Headache	8 (11.4)	4 (18.2)	4 (8.3)	0.249
Chest tightness	6 (8.6)	1 (4.5)	5 (10.4)	0.657
Dyspnoea	10 (14.3)	1 (4.5)	9 (18.8)	0.154
Dizziness	10 (14.3)	6 (27.3)	4 (8.3)	0.061
Fever duration, median (IQR), day	6.00 (2.25–8.75)	4.00 (2.00–6.00)	6.00 (3.00–10.00)	**0.003**
Systolic pressure, median (IQR), mmHg	125.00 (119.00–135.00)	125.00 (118.25–132.75)	125.00 (119.00–135.00)	0.191

Bold indicates the difference is statistically significant

Data are median (IQR), *n* (%), or *n*/*N* (%), where N is the total number of patients with available data. *P* values compare patients with and without pneumonia using *χ*^2^ test, Fisher’s exact test, or student *t* test

*COVID-19* coronavirus disease 2019, *IQR* interquartile range, *BMI* body mass index

**Table 2:** Comparison of laboratory findings at the admission between COVID-19 patients with and without pneumonia

	All enrolled patients (*n* = 70)	Patients without pneumonia (*n* = 22)	Patients with pneumonia (*n* = 48)	*P*
Blood glucose, mmol/L	5.40 (4.90–6.70)	5.00 (4.73–5.65)	5.70 (5.13–7.00)	0.715
Blood lipid, mmol/L	1.16 (0.77–1.60)	1.28 (0.72–1.60)	1.15 (0.79–1.62)	0.617
PaO_2_, mmHg	85.00 (74.00–113.50)	81.00 (73.25–136.25)	86.00 (74.50–113.50)	0.711
PaCO_2_, mmHg	37.00 (34.25–40.75)	39.00 (37.25–41.75)	37.00 (34.00–40.00)	0.945
SaO_2_, mmHg	97.00 (96.00–99.00)	96.00 (96.00–99.00)	97.00 (96.00–99.00)	0.838
ABE, mmol/L	1.65 (0.40–2.28)	2.45 (–0.13–3.08)	1.45 (0.40–2.10)	0.738
WBC, 10^9^/L	4.85 (3.81–6.74)	5.36 (4.03–6.54)	4.44 (3.58–7.40)	0.790
Neutrophils, %	61.20 (53.35–75.53)	55.60 (50.88–66.13)	64.45 (55.80–81.58)	**0.001**
Lymphocyte, %	29.45 (17.77–36.23)	33.10 (24.93–38.73)	27.95 (12.74–34.88)	**0.006**
CRP, mg/L	7.00 (1.64–25.24)	4.20 (1.00–28.65)	9.31 (2.06–24.11)	0.341
IL-6, pg/ml	8.06 (4.77–24.63)	5.87 (4.85–11.58)	13.50 (4.30–28.63)	**0.016**
Procalcitonin, ng/ml	0.05 (0.03–0.07)	0.04 (0.03–0.06)	0.05 (0.04–0.09)	0.441
ESR, mm/60 min	13.00 (4.00–39.00)	10.00 (4.00–28.00)	14.50 (4.25–41.00)	0.371
Lactate, mmol/L	1.97 (1.61–3.31)	2.03 (1.50–2.95)	1.89 (1.61–3.45)	**0.040**
Lymphocytes, /μl	1137.50 (766.50–1457.75)	1376.00 (1075.00–1934.75)	1060.50 (580.00–1376.25)	**0.004**
T lymphocyte, %	69.00 (61.50–77.50)	71.00 (64.50–77.25)	68.00 (59.00–78.00)	0.351
T lymphocyte count, /μl	802.00 (455.00–1069.00)	975.00 (685.00–1365.00)	731.00 (377.25–1019.50)	**0.004**
CD4^+^ lymphocyte, %	37.00 (30.75–41.25)	38.50 (33.00–43.25)	35.50 (30.25–41.00)	0.449
CD4^+^ lymphocyte count, /μl	425.00 (257.75–573.50)	540.00 (364.00–724.00)	377.00 (196.00–513.00)	**0.002**
CD8^+^ lymphocyte, %	30.00 (24.00–37.00)	28.00 (24.00–32.75)	31.00 (24.00–38.00)	0.739
CD8^+^ lymphocyte count, /μl	366.00 (197.50–505.00)	417.00 (295.00–545.00)	326.50 (137.00–490.50)	0.059
< 190	15/65 (23.1)	1/19 (5.3)	14/46 (30.4)	**0.049**
190–1140	50/65 (76.9)	18/19 (94.7)	32/46 (69.6)	
B lymphocyte, %	12.00 (9.75–17.00)	12.00 (9.00–13.75)	12.00 (10.00–19.25)	0.082
B lymphocyte count, /μl	126.50 (85.00–184.75)	141.50 (103.50–224.50)	122.00 (66.00–169.25)	0.068
< 90	19/66 (28.8)	4/18 (22.2)	15 (31.1)	0.471
90–660	47/66 (71.2)	14/18 (77.8)	33 (68.8)	
NK lymphocyte, %	14.00 (10.00–23.00)	13.00 (8.75–27.25)	14.50 (10.00–23.00)	0.754
NK lymphocyte count, /μl	155.50 (74.75–242.00)	159.50 (131.50–322.25)	142.00 (65.75–211.00)	0.343
CD4/CD8	1.16 (0.93–1.58)	1.41 (1.07–1.59)	1.13 (0.85–1.53)	0.566
NLR	2.08 (1.46–3.76)	1.68 (1.46–2.73)	2.25 (1.46–6.18)	**0.001**

Bold indicates the difference is statistically significant

Data are median (IQR), *n* (%), or *n*/*N* (%), where N is the total number of patients with available data. *P* values compare patients with and without pneumonia using *χ*^2^ test, Fisher’s exact test, or student *t* test

*COVID-19* coronavirus disease 2019, *ABE* actual base excess, *CRP* C-reactive protein, *ESR* erythrocyte sedimentation rate, *IL-6* interleukin-6, *NLR* neutrophils/lymphocytes ratio, *PaCO*_*2*_ partial pressure of carbon dioxide in artery, *PaO*_*2*_ partial pressure of oxygen, *PH* hydrogen ion concentration, *SaO*_*2*_ arterial oxygen saturation, *WBC* white blood cell

### Disease course

Of the 70 patients, 67 received interferon spray, 61 received antiviral treatments with lopinavir/ritonavir and or arbidol. In 69 patients (except the one co-infected with HIV and HBV), the mean (95% *CI*) duration from disease onset to being admitted was 7.88 (6.29–9.48) days [5.91 (3.69–8.13) days in pneumonia-free patients and 8.81 (6.71–10.90) in pneumonia patients] and the mean duration from illness onset to being discharged from hospital was 26.30 (23.47–29.12) days [21.09 (16.37–25.81) days in pneumonia-free patients and 29.23 (25.91–32.55) days in pneumonia patients]. Thus, disease course of COVID-19 was approximately 3–4 weeks.

### Factors predicting disease progression

During hospital stay, two cases with mild-type COVID-19 progressed to be common-type COVID-19, 11 severe-type patients deteriorated to be extremely severe-type COVID-19. Of the 11 patients, three died of COVID-19. The 13 cases were categorized as having progressive diseases. Cox regression analysis including the parameters differed between patients with pneumonia and those without indicated that older age and circulating levels of IL-6, CRP, procalcitonin, and lactate at the 1st day after admission to hospital significantly predicted the progression of COVID-19 (Table 3).

**Table 3:** Cox regression analyses for factors predicting the progression of COVID-19

Covariate	HR (95% *CI*)	*P*
Age, years	**1.038 (1.002–1.075)**	**0.039**
Hypertension	2.295 (0.765–6.881)	0.138
Fever	1.278 (0.161–10.146)	0.816
Cough	3.336 (0.734–15.160)	0.119
C-reactive protein, mg/L	**1.018 (1.005–1.030)**	**0.004**
Procalcitonin, ng/ml	**1.356 (1.092–1.684)**	**0.006**
T lymphocyte count, /μl	0.999 (0.997–1.000)	0.119
CD4^+^ lymphocyte count, /μl	0.998 (0.995–1.001)	0.148
CD8^+^ lymphocyte count, /μl	0.998 (0.994–1.001)	0.192
B lymphocyte count, /μl	0.993 (0.984–1.002)	0.133
NK lymphocyte count, /μl	0.996 (0.989–1.002)	0.205
IL-6, pg/ml	**1.021 (1.004–1.039)**	**0.014**
Lactate, mmol/L	**1.023 (1.004–1.043)**	**0.018**
NLR	0.995 (0.904–1.095)	0.921
CD4^+^/total T lymphocyte	1.089 (0.041–29.038)	0.959
CD8^+^/total T lymphocyte	1.121 (0.019–67.083)	0.956

Bold indicates the difference is statistically significant

*COVID-19* coronavirus disease 2019, *IL-6* interleukin-6, *NLR* neutrophils/lymphocytes ratio, *HR* hazard ratio, *CI* confidence interval

**Fig. 1:**
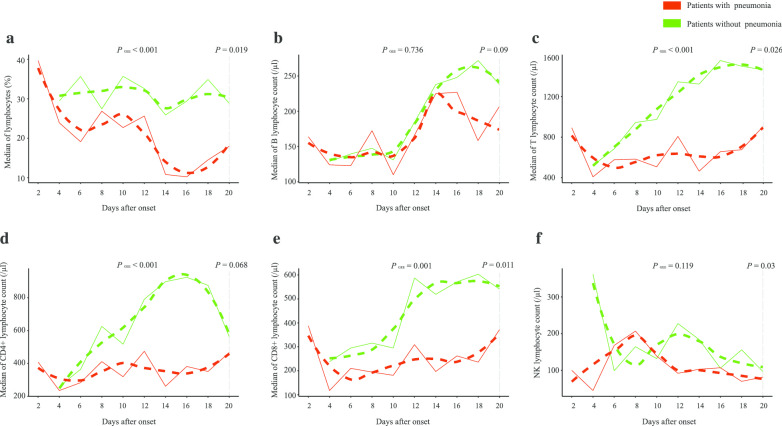
Dynamic curves of lymphocytes and its subsets between COVID-19 patients with pneumonia and those without pneumonia. Daily medians of laboratory parameters within the first 20 days were applied to construct the dynamic curves. *P*_GEE_ indicated statistic difference in the daily medians of each laboratory parameter between patients with and without pneumonia during the 20 consecutive days. *P* value indicates the difference in each laboratory parameter between patients with and without pneumonia at the 20th day since illness onset (Mann–Whitney U test)

**Fig. 2:**
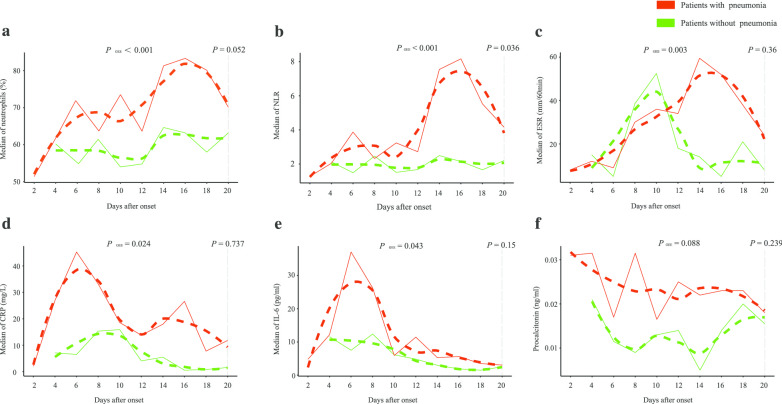
Dynamic curves of other inflammatory factors between COVID-19 patients with pneumonia and those without pneumonia. Daily medians of the parameters within the first 20 days were applied to construct the dynamic curves. *P*_GEE_ indicated statistic difference in the daily medians of each laboratory parameter between patients with and without pneumonia during the 20 consecutive days. *P* value indicates the difference in each laboratory parameter between patients with and without pneumonia at the 20th day since illness onset (Mann–Whitney U test). *CRP* C-reactive protein; *ESR* erythrocyte sedimentation rate; *IL-6* interleukin-6; *NLR* neutrophils/lymphocytes ratio

We re-categorized patients who developed pneumonia and then evaluated the dynamics of clinical and laboratory parameters during hospital stay between COVID-19 patients with pneumonia and those without pneumonia. Of the parameters evaluated, 9 showed significantly different trends between COVID-19 patients with pneumonia and those without. During the course of COVID-19, neutrophil proportion (*P*_GEE_ < 0.001), NLR (*P*_GEE_ < 0.001), and serum levels of CRP (*P*_GEE_ = 0.024), ESR (*P*_GEE_ = 0.003), and IL-6 (*P*_GEE_ = 0.043) were significantly higher in pneumonia patients than pneumonia-free patients, which were quite in contrast to lymphocyte proportion (*P*_GEE_ < 0.001), T lymphocyte count (*P*_GEE_ < 0.001), CD4^+^ T lymphocyte count (*P*_GEE_ < 0.001), and CD8^+^ T lymphocyte count (*P*_GEE_ = 0.001). B lymphocyte count did not change between patients with pneumonia and those without. At the 20th day after illness onset, the dynamic curves of neutrophil proportion, CD4^+^ T lymphocyte count, and the levels of CRP, ESR, IL-6, and procalcitonin between patients with pneumonia and those without tended to meet (*P* > 0.05); however, the two curves of CD8^+^ T lymphocyte counts kept separated (*P* < 0.05) (Figs. 1 and 2).

## Discussion

In this study, most patients had clear exposure history, indicating close contact via droplets was the major way of SARS-CoV-2 transmission in low epidemic regions. The mean incubation of SARS-CoV-2 infection was 8.67 days. A previous study carried out in Wuhan indicated that the mean incubation was 5.2 days [2]. Patients identified at the early stage of the outbreak in Wuhan mostly had severe pneumonia and might be exposed to higher viral concentration in closed spaces like the affected hospitals. As asymptomatic carriers might transmit SARS-CoV-2, person-to-person transmission of SARS-CoV-2 should occur earlier than do SARS-CoV [12, 13]. Quarantine of close contactors and social distancing are necessary during COVID-19 pandemic. The incubation duration from this study should be important in quarantining close contactors.

We determined that the mean duration from onset to discharge was 26.30 days. The course of COVID-19 is approximately 3–4 weeks, which is longer than severe acute respiratory syndrome (SARS) (approximately 2 weeks) [14]. The mean duration of SARS-CoV-2 shedding might be 20 days, which is not shorter than SARS-CoV [15]. A just finished clinical study in Shanghai, China, did not show any therapeutic effect of lopinavir/ritonavir or abidol on accelerating the clearance of SARS-CoV-2 [16]. These evidences imply that the interaction of host immunity with SARS-CoV-2 infection, rather than the current antiviral regimen with lopinavir/ritonavir and abidol, alters the course of COVID-19.

To identify the factors that can indicate progressive COVID-19, we characterized the differences in clinical features and laboratory findings between COVID-19 patients with pneumonia and those without pneumonia firstly. It was found that older age and underlying hypertension were significantly associated with the risk of developing pneumonia. SARS-CoV-2 infection in elders with underlying medical conditions including hypertension often results in serious consequences, which is quite in accordance with SARS and Middle East respiratory syndrome (MERS) [13]. Specific prophylaxis should be given to this vulnerable population. Higher proportion of neutrophil, lower proportion of lymphocytes, lower T cell count, and higher serum level of IL-6 were significantly associated with the risk of developing pneumonia. Unlike those without pneumonia, one-third of COVID-19 patients with pneumonia had very low account (< 190/µl) of CD8^+^ T cells. These data suggest that CD8^+^ T cell exhaustion and IL-6-based inflammation play an important role in COVID-19 progression. As some COVID-19 patients progress rapidly [17], we evaluated the effect of factors including those differed between COVID-19 patients with pneumonia and those without in the cross-sectional study on disease progression in a prospective study. It was identified that serum IL-6, CRP, procalcitonin, and lactate at the time of being admitted to hospital predicted poor outcomes of COVID-19. IL-6 is capable of inhibiting the T cell-mediated immunity, and high level of IL-6 in the acute stage is associated with lung lesions in SARS patients [18]. CRP, an acute inflammatory protein, plays important roles in inflammatory processes and host responses to infection [19]. Procalcitonin reflects bacterial infections [20]. High level of procalcitonin indicates the possibility of secondary bacterial infection. Superinfections with bacteria during hospitalization are significantly related to worse outcomes of COVID-19 patients [21]. Lactate, a natural by-product of aerobic glycolysis, can promote the switch of CD4^+^ T cells to an IL-17^+^ subset and impairs the cytolytic capacity of CD8^+^ T cells [22]. Thus, IL-6 and lactate might bridge SARS-CoV-2 infection and alveolar cell injury via inducing tissue-damaging inflammation, and impairs the CD8^+^ T cell-mediated antiviral immunity.

For the first time, the dynamics of immune and inflammatory factors were evaluated during hospitalization of COVID-19 patients. Neutrophil proportion, NLR, IL-6, ESR, CRP, and procalcitonin were generally higher in the pneumonia patients than in the pneumonia-free patients, in contrast to CD8^+^ and CD4^+^ T lymphocyte counts. Interestingly, CD4^+^ T lymphocyte counts recover rapidly, whereas CD8^+^ T lymphocyte cells in pneumonia patients did not recover at the 20th day, indicating that recovery of CD8^+^ T cell-mediated immunity needs a longer time. Thus, the discharged patients need extensive medical care. A recent study carried out in Germany has demonstrated that reappearance of effector T cells is associated with COVID-19 recovery [23]. Thus, the recovery of T cells count, especially CD8^+^ T cell count should be extensively monitored after being discharged. According to the results of this study, we suggest that patients with severe COVID-19 or patients bearing the risk of developing severe COVID-19 may benefit from immunotherapy with autologous CD8^+^ T lymphocytes and/or cytokine up-regulating the number and function of CD8^+^ T lymphocytes. Although interferon was suggested for the treatment of COVID-19 [24, 25], immunotherapy to up-regulate CD8^+^ T lymphocytes has not been established. Thus, this study may help in designing immunotherapy for COVID-19.

Our study has several limitations. First, the sample size was small, which limited further statistical analysis. Second, the course of severe patients might be prolonged by therapeutic regimens including corticosteroids [26].

## Conclusions

Old age, hypertension, higher circulating levels of neutrophils, IL-6, and NLR and lower levels of T lymphocytes, CD4^+^ lymphocytes, and CD8^+^ T cells were significantly associated with the risk of developing pneumonia. In patients with COVID-19, disease progression is significantly associated with older age and higher circulating levels of CRP, procalcitionin, IL-6, and lactate. T lymphocyte, especially CD8^+^ T cell-mediated immunity is important in recovery of COVID-19. This study may help not only in predicting disease progression but also in designing immunotherapy for COVID-19.

## Supplementary information

**Additional file 1: Figure 1.** Chronology of symptom onset of the family clustering COVID-19 cases in Beijing and their contacts in Wuhan, Hubei province, China.

**Additional file 2: Figure 2.** Consecutive COVID-19 patients admitted to the study hospital. Wuhan-direct, the patients who traveled to Wuhan or came from Wuhan within 14 days before illness onset; Wuhan-indirect, the patients who ever exposed to a late—diagnosed patient from Wuhan within 14 days before illness onset; and no explicit contact history, the patients did not have the above 2 situations. Fourteen days after Wuhan quarantine, 7 patients were admitted and 3 of the 7 were from other countries.

## Data Availability

The datasets used and analyzed during the current study are available from the corresponding author on reasonable request.

## Abbreviations

COVID-19: Coronavirus disease 2019
SARS-CoV-2: Severe acute respiratory syndrome coronavirus 2
CRP: Serum C-reactive protein
IL-6: Interleukin-6
ESR: Erythrocyte sedimentation rate
PaO_2_: Arterial partial pressure of oxygen
FiO_2_: Fraction of inspired oxygen
NLR: Neutrophil/lymphocyte ratio
HIV: Human immunodeficiency virus
HBV: Hepatitis B virus
GEE: Generalized Estimating Equations
