# Supplementary figures and images for: Increased circulating level of interleukin-6 and CD8+ T cell exhaustion are associated with progression of COVID-19
Source: Infect Dis Poverty. 2020 Nov 25;9:161. doi: 10.1186/s40249-020-00780-6 (PMC7686818; doi:10.1186/s40249-020-00780-6)

A

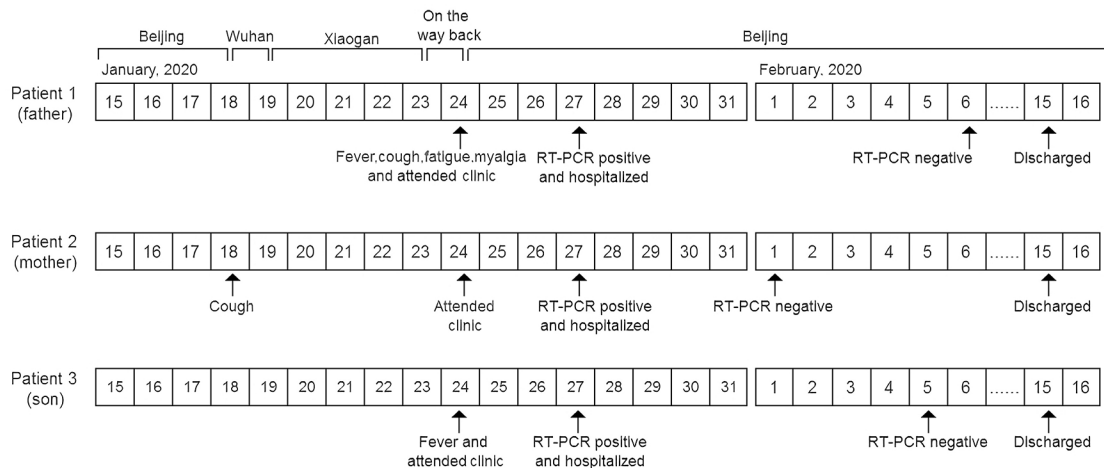

B

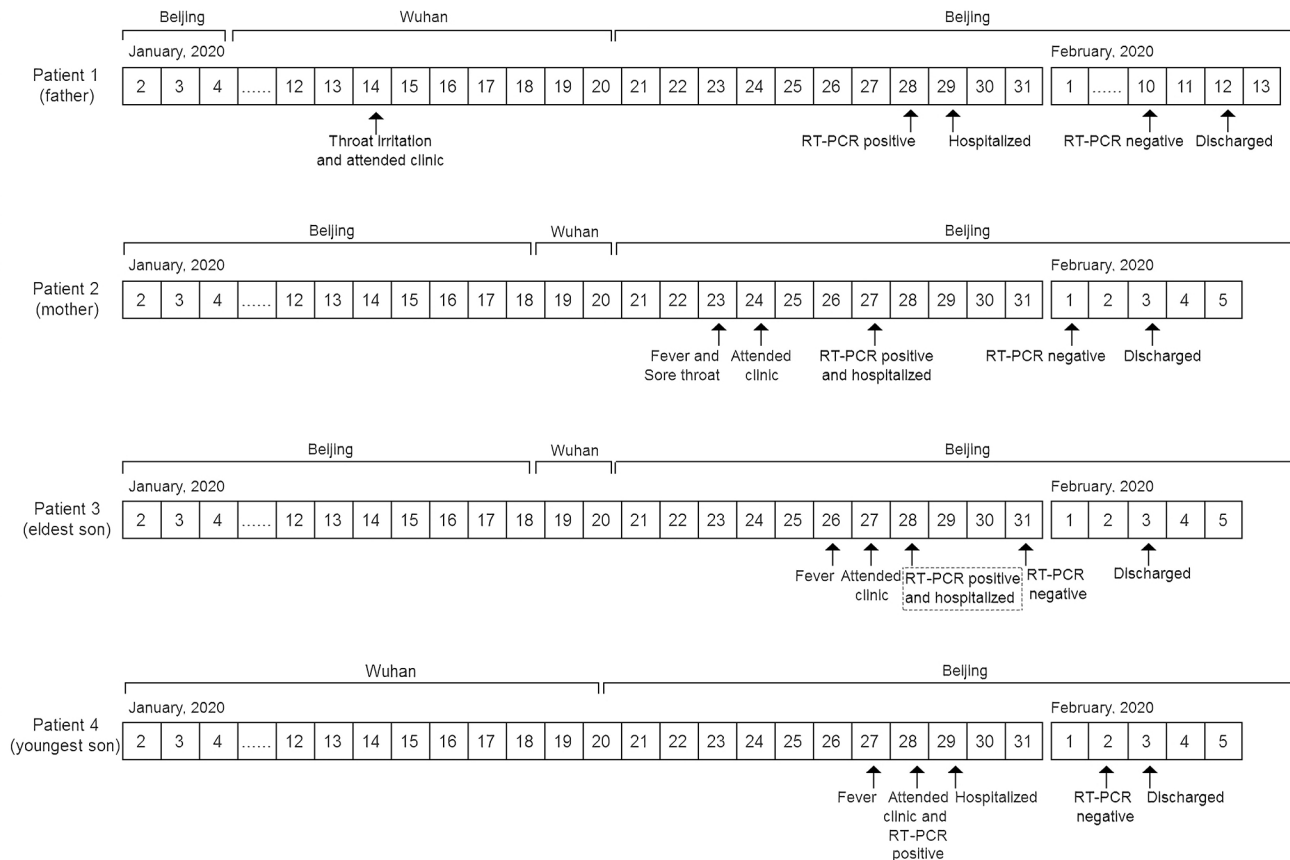

Supplement: Supplementary file 1 — Additional file 1: Figure 1. Chronology of symptom onset of the family clustering COVID-19 cases in Beijing and their contacts in Wuhan, Hubei province, China. [file 40249_2020_780_MOESM1_ESM.pdf]

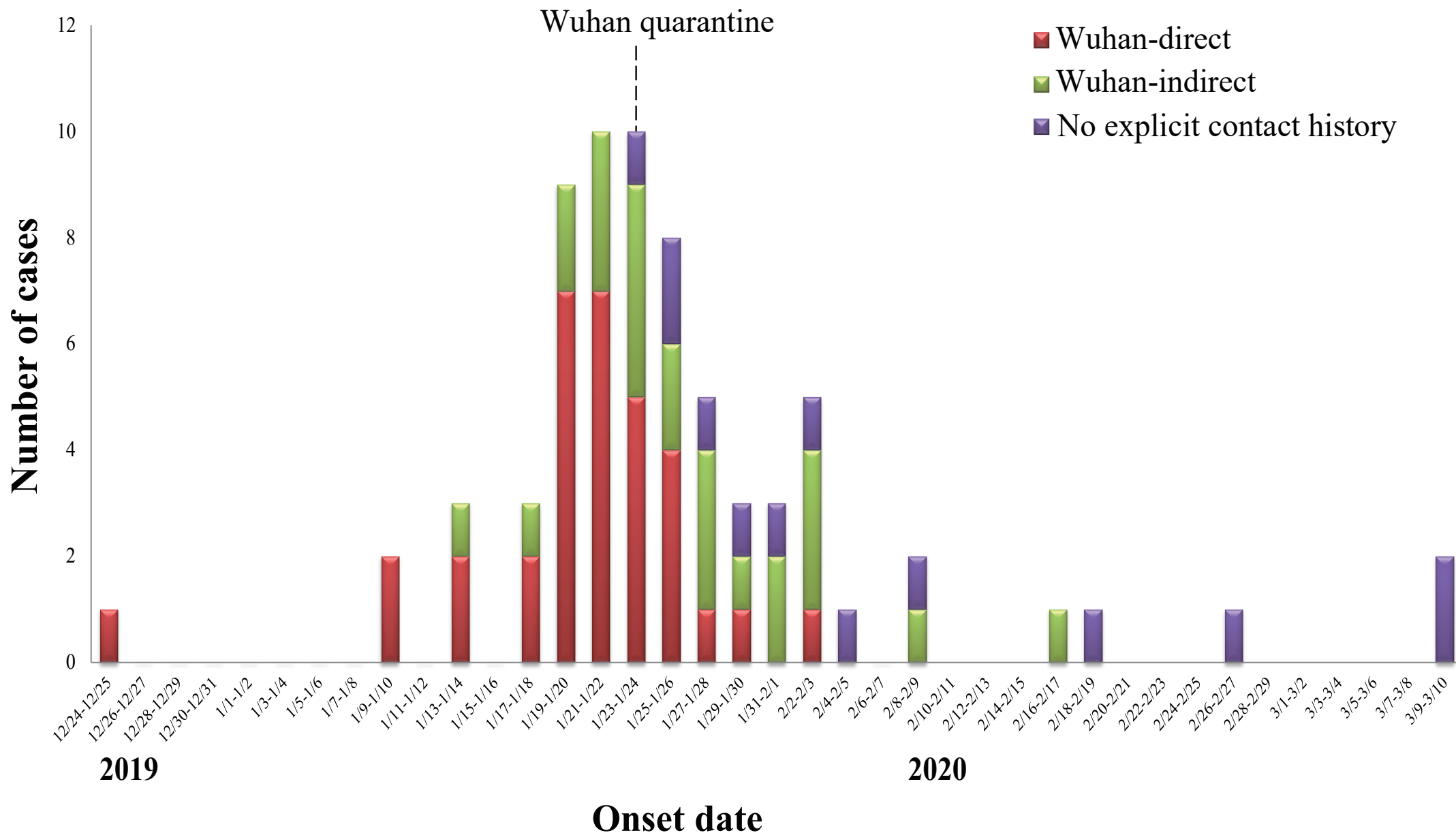

Supplement: Supplementary file 2 — Additional file 2: Figure 2. Consecutive COVID-19 patients admitted to the study hospital. Wuhan-direct, the patients who traveled to Wuhan or came from Wuhan within 14 days before illness onset; Wuhan-indirect, the patients who ever exposed to a late—diagnosed patient from Wuhan within 14 days before illness onset; and no explicit contact history, the patients did not have the above 2 situations. Fourteen days after Wuhan quarantine, 7 patients were admitted and 3 of the 7 were from other countries. [file 40249_2020_780_MOESM2_ESM.pdf]
